# Impact of PermaNet 3.0 on entomological indices in an area of pyrethroid resistant *Anopheles gambiae* in south-western Nigeria

**DOI:** 10.1186/1756-3305-7-236

**Published:** 2014-05-22

**Authors:** Samson T Awolola, Adedapo O Adeogun, Judith B Olojede, Adedayo O Oduola, Isaac O Oyewole, Chioma N Amajoh

**Affiliations:** 1 Molecular Entomology and Vector Control Research Laboratory, Nigerian Institute of Medical Research, PMB 2013 Yaba Lagos, Nigeria; 2 Department of Zoology, University of Ibadan, Ibadan, Nigeria; 3 Department of Zoology, University of Ilorin, Ilorin, Nigeria; 4 Department of Biological Sciences, Babcock University, Ilishan Remo, Nigeria; 5 National Malaria Control Program, Federal Ministry of Health, Abuja, Nigeria

**Keywords:** PermaNet 3.0, Pyrethroid resistance, *Anopheles gambiae*

## Abstract

**Background:**

PermaNet® 3.0 is an insecticide synergist-combination long-lasting insecticidal net designed to have increased efficacy against malaria vectors with metabolic resistance, even when combined with *kdr*. The current study reports on the impact of this improved tool on entomological indices in an area with pyrethroid-resistant malaria vectors in Nigeria.

**Methods:**

Baseline entomological indices across eight villages in Remo North LGA of Ogun State provided the basis for selection of three villages (Ilara, Irolu and Ijesa) for comparing the efficacy of PermaNet® 3.0 (PN3.0), PermaNet® 2.0 (PN2.0) and untreated polyester nets as a control (UTC). In each case, nets were distributed to cover all sleeping spaces and were evaluated for insecticidal activity on a 3-monthly basis. Collection of mosquitoes was conducted monthly via window traps and indoor resting catches. The arithmetic means of mosquito catches per house, entomological inoculation rates before and during the intervention were compared as well as three other outcome parameters: the mean mosquito blood feeding rate, mean mortality and mean parity rates.

**Results:**

*Anopheles gambiae s.l.* was the main malaria vector in the three villages, accounting for >98% of the *Anopheles* population and found in appreciable numbers for 6–7 months. Deltamethrin, permethrin and lambdacyhalothrin resistance were confirmed at Ilara, Irolu and Ijesa. The *kdr* mutation was the sole resistance mechanism at Ilara, whereas *kdr* plus P450-based metabolic mechanisms were detected at Irolu and Ijesa. Bioassays repeated on domestically used PN 2.0 and PN 3.0 showed persistent optimal (100%) bio-efficacy for both net types after the 3^rd^, 6^th^, 9^th^ and 12^th^ month following net distribution. The use of PN 3.0 significantly reduced mosquito densities with a ‘mass killing’ effect inside houses. Households with PN 3.0 also showed reduced blood feeding as well as lower mosquito parity and sporozoite rates compared to the PN 2.0 and the UTC villages. A significant reduction in the entomological inoculation rate was detected in both the PN 2.0 village (75%) and PN 3.0 village (97%) post LLIN-distribution and not in the UTC village.

**Conclusion:**

The study confirms the efficacy of PN 3.0 in reducing malaria transmission compared to pyrethroid-only LLINs in the presence of malaria vectors with P450-based metabolic- resistance mechanisms.

## Background

The use of long-lasting insecticidal nets (LLINs) and indoor residual spraying (IRS) remains the mainstay for malaria prevention. However, the development of resistance by *Anopheles* mosquitoes to all classes of WHO-recommended adult insecticides, particularly pyrethroids, is a serious concern and threat to malaria control [1,2]. Of the four classes of insecticides (pyrethroid, organochlorine, organophosphate and carbamate) currently recommended for malaria vector control, only pyrethroid is currently approved for LLINs because of its safety, residuality and cost effectiveness. A key issue is to maintain the effectiveness of these vector control tools in an era of growing resistance.

In Nigeria, the first case of pyrethroid resistance in malaria vectors was reported in 2002 [3,4]. Evidence of resistance has since then increased, and is now reported in 16 States affecting the two most important malaria vectors: *Anopheles gambiae s.s*. and *Anopheles arabiensis*[5-7]. While other causal factors of resistance have been identified, such as agricultural usage of insecticides, the significant increase in insecticide-based malaria vector control in the last 10 years has likely exerted significant insecticide selection pressure on *Anopheles* populations in the country. Two main mechanisms of resistance (target-site *kdr* mutations and metabolic alterations) have been identified in different areas [8] but resistance data are still limited. The reality and impact of resistance at the program level is unfolding and it is believed that the loss of pyrethroid effectiveness will lead to increases in preventable deaths particularly in the most vulnerable groups. Consequently, the World Health Organization recommends immediate and pre-emptive action to delay resistance [9]. This requires tools with high efficacy. Current WHO-recommended strategies for insecticide resistance management include: (i) rotational use of insecticides with different modes of action, (ii) combination of interventions, (iii) mosaic spraying, and (iv) application of mixtures of insecticides [9]. Unfortunately, these strategies are most appropriate for IRS. For LLINs, tools with improved efficacy against resistant mosquitoes are limited because pyrethroid is the only insecticide class currently used on LLINs.

Two next generation LLINs have been developed to provide additional efficacy against pyrethroid-resistant mosquitoes through a combination of a pyrethroid with the synergist piperonyl butoxide (PBO), known to affect resistant mosquitoes by inhibiting metabolic enzymes responsible for breaking down pyrethroid molecules. The first combination LLIN was PermaNet® 3.0, which received a WHO interim recommendation as an LLIN in 2008 [10]. This LLIN combines deltamethrin coated polyester side panels and deltamethrin with PBO incorporated in the polyethylene roof. More recently, OlysetPlus® received a WHO interim recommendation in 2012 [11]. OlysetPlus® combines permethrin with PBO incorporated in the polyethylene roof and sides.

In line with the policy of the Nigerian National Malaria Control Program prior to the introduction of an improved vector control tool, a village-wide impact study of PermaNet® 3.0 against pyrethroid resistant malaria vectors was conducted in relation to a pyrethroid only LLIN (Olyset nets) in 2010 [12]. Data from this study have shown the potential of PN 3.0 in controlling resistant malaria vectors when compared to a pyrethroid only LLIN (Olyset nets). The present study was designed to compare the efficacy of PermaNet® 3.0 to another standard pyrethroid only LLIN (PermaNet® 2.0) commonly used in Nigeria. Product acceptance and user perception of efficacy were also investigated.

## Methods

### Study area

The study was carried out in Remo North Local Government Area of Ogun State, South Western Nigeria. The climate of the area is characteristic of the forest zone with two distinct seasons. The rainy season from April to October and dry season from November to March. The mean annual rainfall is 2000 mm with a mean relative humidity of 78% [13]. The mean temperature is 24°C during the wet and 30°C during the dry season. The area consists of fifteen agrarian communities of approximately 5000 people. Around these communities are small cocoa and palm tree plantations in addition to small vegetable gardens. Herds of cattle and goat kept by nomadic Fulani herdsmen are common in the area. Housing structures consist of both traditional houses (20–35%: mud wall with thatched roof) and modern houses (60–65%: brick houses with corrugated iron roof). The inhabitants are mainly of the Yoruba ethnic group with similar culture and traditions. Malaria is endemic with perennial transmission associated with *Anopheles gambiae s.s*[14]. As a result of baseline insecticide resistance data collected in the area, three villages between 3–5 km apart: Ilara (06° 55.186’ N; 003° 48.200’ E), Irolu (06° 54.423’ N; 003° 44.737’E) and Ijesa (06° 54.659’ N; 003° 46.160’ E) were selected for comparing the efficacy of PN 3.0 with PN 2.0 and the UTC. The villages are similar in term of size, housing structure and population. However, the most important criteria for their selection is the presence of insecticide resistance.

### Baseline data

#### Insecticide susceptibility test and synergist study

Insecticide susceptibility tests were conducted on mosquitoes collected from the 8 villages in March 2012. Two to three day old adult *An. gambiae s.l.* reared from larval collection in each village were identified morphologically [15,16] and were exposed to permethrin (0.75%), deltamethrin (0.05%), lambdacyhalothrin (0.05%) and DDT (4%) for 1 h, following the standard WHO protocol [17]. For each village, 100–140 female *Anopheles* (5–7 replicates of 20 mosquitoes) were used per test paper. Three villages (Irolu, Ijesa and Ilara) had the highest rate of insecticide resistance. The population *of An. gambiae s.l.* that survived the insecticide exposure in these three villages was divided into two: (1) the first subset was analyzed together with dead mosquitoes to species level using PCR [18] and also for the presence of the *kdr* mutation using allele-specific PCR diagnostic tests designed for the West African *kdr* mutation [19]; (2) the second subset was induced to lay eggs in the insectary and F1 progeny were used for synergist and biochemical analyses as previously described [20]. In brief, PBO was tested for synergistic activity with permethrin or deltamethrin; mortality was compared between mosquitoes exposed and unexposed to PBO to determine the role of metabolic degradation as a mechanism for pyrethroid resistance. To investigate the relative role of specific metabolic pathways inhibited by this synergist, enzyme assays were also carried out on live mosquitoes to measure esterase, glutathione S-transferase (GST) and cytochrome P450 monooxygenase activity [20-22]. All mosquitoes tested were identified to species level by PCR [18].

#### Adult mosquito collection

Adult mosquitoes were sampled once prior to net distribution in 35 houses in each of Irolu, Ijesa and Ilara using exit trap and indoor resting collections. The baseline data enabled the determination of vector species, indoor resting densities, blood feeding rates, mortality and determination of sporozoite rates prior to net distribution.

#### Mosquito nets and treatment arms

PermaNet 2.0 and PermaNet® 3.0 were provided by Vestergaard Frandsen, Switzerland with a production date of October 2010. Untreated polyester nets were procured from a local market in Lagos, Nigeria. Each village was randomly assigned to a treatment arm: PermaNet® 3.0 to Irolu, PermaNet® 2.0 to Ijesa and untreated nets to Ilara. Following house enumeration and completion of households records, 137 PN 3.0 were distributed at Irolu to cover all sleeping spaces, 147 PN 2.0 were distributed at Ijesa resulting in 100% coverage of all sleeping spaces and 150 untreated polyester net were provided at Ilara, also covering all sleeping places. The nets were distributed on the same day (15^th^ March 2012) in the three villages. Nets were given a unique code and a “net master list” developed for each village for follow-up. Householders were provided with basic information on correct net usage. Prior to the distribution, existing nets at Irolu and Ijesa were collected and replaced with test nets. Existing nets in the control village were left with net owners. Before the commencement of the study, village group meetings were held and people were educated on the objectives of the study. Householders were provided with basic information on correct net usage.

### Net selection for *in situ* bio-assay cone test

WHO guidelines for phase 3 trials [23] recommend that at least 30 nets per experimental arm are tested in bioassays. Therefore, 35 households were selected randomly from each treatment arm to account for potential drop-outs later in the study. From each of these households, a room where one man slept under the net (one room housing a single man) was selected. The same nets were tested at baseline (March 2012) and were then evaluated during each quarterly bioassay test (June 2012, September 2012, December 2012 and March 2013).

Bio-efficacy was assessed using the reference Kisumu susceptible laboratory strain of *An. gambiae s.s.* in a standard WHO cone test [23]. For all net types, four side panels and the roof panel of each net was tested. One cone test was conducted per side panel, with five 2–3 day old non-blood-fed female mosquitoes used per cone for a total of 25 mosquitoes tested on each net.

### Entomological assessment

#### Mosquito collection and identification

Adult mosquitoes were sampled from 35 houses with nets previously selected for quarterly cone bioassay. One room housing a single man was used; collections were made once prior to net distribution in March 2012, and thereafter once per month for 12 months (April 2012 to March 2013). The same houses were used for the duration of the study. After net distribution, mosquitoes were collected on the 15^th^day of each month by a team of entomologists per village. The three teams were randomly rotated and allocated to a village each month. Mosquito densities were measured by the following methods:

(i) Window exit trap collection: 35 window traps were used in the selected houses in each village. Traps were in place by 18.00 hrs and mosquitoes were collected from it the following morning (06.00 hr). Locally sourced field workers including householders in whose dwellings the traps were placed were trained to support the entomology technicians for mosquito collection. They were instructed and shown how to block the exit trap by 06.00 hrs and collect live and dead mosquitoes from the window traps. Mosquitoes were placed into pre-labelled tubes with the number, name of the site and name of the householder marked. Alive and dead mosquitoes were placed in different tubes for further analysis.

(ii) Indoor resting collection: Sampling took place in rooms without window traps, and the same houses were used for each of the monthly samples with the houses being sampled in the same order each month. 35 sleeping rooms with LLINs selected for periodic cone bioassay were included in indoor resting catches. Resting catches were carried out using a standard methodology (a 10 minute search) between 06.00-08.00 hrs using a flash light [24]. The number of mosquitoes collected in each house and their physiological status (unfed, blood fed, gravid) were recorded and *Anopheles* mosquitoes were identified using morphological keys. All *An. gambiae s.l.* were preserved individually on desiccated silica gel for PCR identification and *kdr* status. Host blood feeding preference was assessed by ELISA tests in the laboratory [25].

#### Parity rate and determination of source of blood meal and Plasmodium infection in mosquitoes by ELISA

Live mosquitoes collected were dissected to determine the parity rate, including all *An. gambiae s.s*. collected at baseline and each month during the 12 months evaluation in the LLIN villages together, with 3590 representing 50% of the total collected in the UTC village post-intervention. The blood meal analysis included all blood fed mosquitoes collected at baseline and in the LLIN villages during 12 months following net distribution together with 2000 (about 50%) blood fed mosquitoes collected from the UTC village over the same period. To estimate the *Plasmodium* infection rate in the mosquito populations, the head and thorax of all female *Anopheles* mosquitoes collected were cut and processed using an ELISA assay [26].

#### Net tracking and household questionnaires

Two methods were used to collect data. Initially, house-to-house surveys for net usage and physical status of nets (identification, counting and measurement of size of holes in the nets) were conducted monthly. Using the net master list, all self-identified heads of households were interviewed. The questionnaires were used to determine people’s perception of the benefits and/or side effects during use of nets. Where nets were no longer available, interviews were conducted once to determine reasons for halted usage. Focus group discussions were conducted after 12^th^ months with the household heads and individuals sleeping under the nets to obtain descriptive information on the households’ perception on the use of LLINs.

#### Determination of chemical content of nets

Five PN 2.0 and five PN 3.0 were randomly collected from net owners and replaced with new nets after the 6^th^ and 12^th^ month of field use. 25 × 25 cm samples were cut from each of the four side panels and the roof panel of each net and were processed for chemical assays according to CIPAC method at an ISO-certified laboratory in Vietnam. A second set of samples (25 × 25 cm) from the same nets were stored at 4°C for reference purposes.

### Data analysis

Data collected were analyzed using the STATA statistical package (STATA Corp LP, USA, version 9.1). Treatment arms and net allocation per village was blinded to the statistician to avoid potential bias. There was a positive skew in distribution of the data with a number of zero counts. A logarithmic transformation was therefore used for an approximation to a normal distribution. Counts of mosquitoes from each village were log transformed [ln (n + 1)] to normalize the data with the geometric mean modified to Williams mean to accommodate zero values [27]. The modified geometric means of mosquito catches per village before and during the intervention were compared as well as three other outcome parameters: the geometric means of mosquito blood feeding, mortality and parity rates amongst PN3.0, PN 2.0 villages and the village with untreated nets. For each entomological parameter comparisons amongst treatment groups were made by chi square tests with the significance level set to p-value <0.05.

Biting rates per room per day were calculated by dividing the total number of blood-fed mosquitoes caught by the number of persons sleeping in the room the night preceding the collection [28]. Entomological inoculation rates were calculated as the product of the sporozoites and man biting rates [28,29].

Survey questionnaires were summarized on excel spread sheets and analysed using an excel database. Comparisons of proportions between categorical variables were performed using a chi square test.

## Results

### Mosquito species and abundance

A total of 13, 030 anophelines were collected during the study, of which 12, 788 (98.1%) were *Anopheles gambiae s,l*., the remainder being *Anopheles nili,* or *An. funestus* with no significant difference in proportion of these species found in the exit trap and room collections in any of the treatment arms. The 12, 788 *An. gambiae s.l*. correspond to 2,015 at baseline and 10,773 during the 12 months following net distribution (Table 1). PCR analysis of the *An. gambiae* s.l. showed that all samples from Ilara were *Anopheles gambiae s.s*. A predominance of *An. gambiae s.s.* was also recorded at Irolu (95% *An. gambiae s.s*, 4.5% *An. arabiensis*) and Ijesa (98.1% *An. gambiae s.s*, and 1.6% *An. arabiensis*). The percentage of *An. gambiae s.s.* during the 12 months post intervention in the three villages was similar to baseline (100% at Ilara, 96% at Irolu and 99% at Ijesa). PCR analysis for the molecular form of *Anopheles gambiae s.s*. identified the collections either as a mix of approximately 80% of the S and 19% of the M form at Ijesa (PN 2.0 village) or as pure collections of the S form at Ilara (UTC village) and Irolu (PN 3.0 village) respectively. This proportion did not change during the 12 months following net distribution.

**Table 1:** **Numbers of *Anopheles gambiae s.l. *** **collected in each village with the average room**

**Site**	**Baseline**		**After**	
	No collected*	**Average room density**	No collected**	**Average density**
**Ilara (UTC)**	568	16.2	7182	17.1
**Irolu (PN 3.0)**	702	20.1	573	1.4
**Ijesa (PN 2.0)**	745	21.3	3018	7.2
Total	2,015		10,773	

Density prior to net distribution and during the following 12 months.

*Number of *Anopheles gambiae s.l.* collected in 35 rooms once prior to net distribution in each village.

**Total number of *Anopheles gambiae s.l.* collected in 35 rooms once per month following nets distribution from April 2012 to March 2013.

### Phenotypic resistance

Using WHO criteria [17], permethrin, deltamethrin, lambdacyhalothrin and DDT resistance were found in the three villages (Ilara, Irolu and Ijesa) during the baseline survey. In addition, DDT and permethrin resistance was found in four other villages in the study area. The 24 h post exposure mortality at baseline for deltamethrin in the three villages was < 64% (Table 2). Twelve months after the intervention, the resistance status of the *Anopheles* populations in the three villages was similar to the pre-intervention level, with the highest resistance still occurring at Irolu (PN 3.0 village; mean 24 h post exposure mortality for all four insecticides of < 63%).

**Table 2:** Summary of main entomological findings for each village at baseline and monthly mean during the 12-months post-intervention period

**Site**		**Villages**					
		**Ilara**		**Irolu**		**Ijesa**	
**Intervention**		**Baseline**	**UTC**	**Baseline**	**PermaNet 3.0**	**Baseline**	**PermaNet 2.0**
WHO susceptibility test*	% Mortality	72.5	76.0	62.5	64.0	66.7	70.0
	*N*	120	100	120	100	120	100
**Density (mean per house)**		16.2	17.1	20.1	1.4	21.3	7.2
**Mean mortality (%)**		0.65	0.9	1.0	55.1	1.7	24.2
**Mean blood feeding rate (%)**		52.1	57.3	47.3	3.9	48.1	19.9
**Overall mean parity rate (%)**		48.7	45.9	48.1	10.7	40.9	22.8
**Overall mean sporozoites rate (%)**		1.76	2.09	2.14	0.87	3.08	2.81
**EIR**		28.5	26.9	43.0	1.1	65.6	20.2
**Resistance mechanisms identified**		*kdr*		*kdr +* metabolic (p450)		*kdr +* metabolic (p450)	

*with deltamethrin (0.05%).

UTC: untreated control.

### Resistance mechanisms

*kdr mutations: Kdr* alleles were detected at a high level in the villages where resistance was confirmed and at a low level where the mosquito population was susceptible to at least one of the four insecticides tested. The *kdr* frequencies in the three villages ranged between 55–78% at baseline and 52-72% after the intervention. The highest values, 78% at baseline and 72% following intervention were recorded at Ilara (UTC village).

### Metabolic alterations

**Figure 1:**
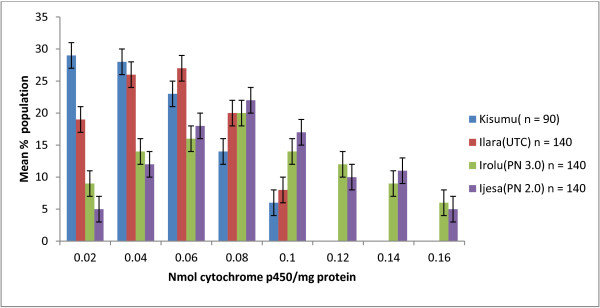
**Mean level of P450 monooxygenase activity in pyrethroid resistant *Anopheles gambiae s.s*. from Ilara, Irolu and Ijesa in relation to the standard reference susceptible Kisumu strain of *Anopheles gambiae s.s.***

Figure 1 shows biochemical analyses indicating that *An. gambiae s.s* from Irolu (PN 3.0 village) and Ijesa (PN 2.0 village) had an increased level (>2 fold) of P450 activity compared with the standard Kisumu strain (Irolu, p = 0.049; Ijesa p = 0.047). The mean P450 activity of *An. gambiae s.s.* from Ilara was similar to that of the Kisumu strain (p = 0.891). There was no significant difference between baseline and post intervention P450 activity for the three villages (P > 0.05). Esterase and GST activities were low in all mosquitoes tested at pre- and post-intervention. The mean esterase activity for mosquitoes from the three villages were similar to that of the Kisumu reference strain (Irolu, p = 0.660, Ijesa, p = 0.723; Ilara, p = 0.755). The mean GST activity for each of the three villages was also similar to that of the reference Kisumu strain indicating that there was no esterase or GST resistance in the mosquitoes from the three villages.

### Bioefficacy of PermaNet 3.0 and PermaNet 2.0

**Figure 2:**
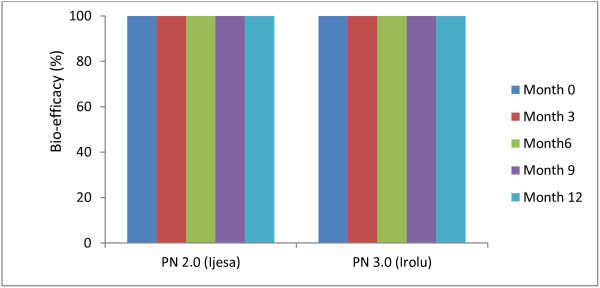
**Bio-efficacy of PermaNet 2.0 and PermaNet 3.0 following 0, 3, 6, 9 and 12 months of field usage, as measured against a susceptible strain of *Anopheles gambiae s.s *** **(Kisumu) in WHO cone bioassays.**

Baseline bioassay conducted on the net samples prior to net distribution showed high efficacy of PN 3.0 and PN 2.0 with 100% mortality against the susceptible Kisumu reference strain of *An. gambiae* s.s. The efficacy remained the same (100%) for both net types after the 3^rd^, 6^th^, 9^th^ and 12^th^ month following net distribution (Figure 2).

### Chemical content of nets of LLINs

The amount of deltamethrin in the LLINs was within the original target dose at 6 months for both the roof and sides of PN3.0 and PN2.0. However, by 12 months the amount of deltamethrin had reduced for PN3.0 and PN2.0 sides, but remained high in the PN3.0 roof. Although the amount of PBO had decreased below the target dose after 6 months of use, there was no further reduction between 6 and 12 months post-distribution (Table 3).

**Table 3:** Chemical content of PermaNet 3.0 and PermaNet 2.0 LLINs after 6 and 12 months of use in Irolu and Ijesa, respectively

**Net type**	**Net section**	**Chemical**	**Units**	**Initial target dose before use**		**After 6 months in use**	**After 12 months in use**
				**Mean**	**Range**	**Mean ± SD**	**Mean ± SD**
PN 3.0	Sides	Deltamethrin	g/kg	2.8	2.1 - 3.5	2.39 ± 0.28	2.67 ± 0.81
	Roof	Deltamethrin	g/kg	4.0	3.0 - 5.0	3.71 ± 0.26	3.63 ± 0.20
		PBO*	g/kg	25.0	18.8 - 31.3	12.0 ± 2.28	12.8 ± 4.34
PN 2.0	Sides	Deltamethrin	g/kg	1.8	1.4 - 2.3	1.60 ± 0.6	1.26 ± 0.42
	Roof					1.78 ± 0.49	1.42 ± 0.47

*Piperonyl butoxide.

### Impact of PN 3.0 and PN 2.0 on malaria transmission indices

#### Vector densities

**Figure 3:**
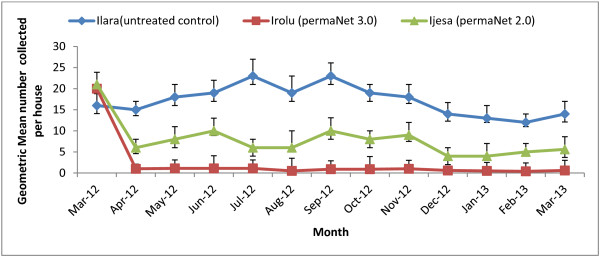
**Geometric mean densities of *An. gambiae s.s*. collected per house at baseline (March 2012) and then monthly for 12-months post intervention at Ilara, Irolu and Ijesa.**

The average number of *Anopheles* found per room, as assessed by exit trap and indoor resting catches at the start of the study in March 2012, was similar in the three villages with a mean of 16.2 at Ilara (UTC village), 20.1 at Irolu (PN 3.0 village) and 21.3 at Ijesa (PN 2.0 village) (Table 1). The numbers in Ilara were elevated at the start of the rainy season in May (Figure 3) and remained so until October before declining to a lower level in February. Here, the malaria vector occurred in large numbers for 6–7 months (May–November) mainly during the wet season with a Williams mean density of 17.1 for the 12 months post-intervention period. On average, a lower density of mosquitoes was detected starting from November to February. This pattern of seasonal abundance was also shown at Ijesa (Figure 3) in spite of the decline in *Anopheles* density following PN 2.0 distribution, but could not be established at the PN 3.0 village because of the significant reduction in mosquito density immediately following net distribution and throughout the following 12 months (Figure 3).

#### Vector mortality, blood feeding and parity rates

The baseline data prior to net distribution showed similar rates of mosquito mortality (0.65–1.5%), blood feeding (47–52%) and parity (41–48%) in the three villages (Table 2).

**Figure 4:**
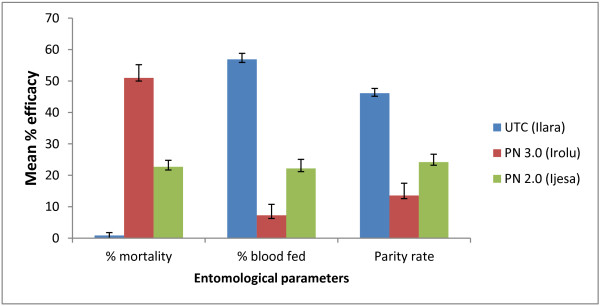
**Geometric mean of percentage mortality, blood feeding and parity rate for *An. gambiae s.s. *** **collected indoor during 12-months post intervention at Ilara, Irolu and Ijesa.**

The use of PN 3.0 at Irolu resulted in high mosquito mortality (Figure 4) with a Williams mean of 50.9% (CI:47.8–58.5) compared to Ijesa (PN 2.0) (mean mortality of 22.7% (CI: 19.8–25.4) and Ilara (UTC control village) (<1% mosquito mortality) (Figure 4). PN 3.0 resulted in a lower blood feeding rate with a mean of 7.3% (CI: 2.8–8.1) compared to Ijesa (PN 2.0) with a mean of 22.2% (CI: 18.4–26.5) and Ilara (UTC) with a mean of 56.9% (CI: 51.2–62.8). The use of PN 3.0 at Irolu also reduced mosquito parity rates (Figure 4) with a mean of 13.6% (CI: 7.6–15.2) compared to a mean of 24.2% in the PN 2.0 village (CI: 19.6–26.8) and 46.1% (CI: 41.1–52.5) in the UTC village. The relatively low parity rate at Irolu is an indication of the high efficacy of PN 3.0 resulting in high mortality of *Anopheles* that had completed a gonotrophic cycle compared to Ijesa (PN 2.0) and the UTC village.

#### Source of mosquito blood meal and vector sporozoite rates

At baseline, 80-85% of mosquito blood meals from the three villages were from humans and the remainder were from cattle or other hosts (Table 2). This remained the same (81.1%) in the UTC village during the post intervention period. In contrast, following LLIN distribution, there was a significant reduction in the number of human blood meals in mosquitoes from the PN 3.0 village (P = 0.042) with a corresponding increase in cattle blood meals (mean 70.4%). There was also a reduction in human blood meals in mosquitoes from the PN 2.0 village when compared to baseline but this difference was not significant.

Results from the sporozoite ELISAs for the three villages are shown in Table 4. At baseline, *Plasmodium falciparum* sporozoite rates in the mosquito population were 1.8% at Ilara (UTC village), 2.1% at Irolu (PN 3.0 village) and 3.1% at Ijesa (PN 2.0 village). The use of PN 3.0 at Irolu resulted in a significant reduction in the sporozoite rate (declined to 0.9%) (P = 0.022). The sporozoite rate in the PN 2.0 and the UTC villages remained statistically similar post-intervention as at baseline (Table 4). The estimated monthly entomological inoculation rate (EIR) before bed net distribution was 28.5 at Ilara, 43.0 at Irolu and 65.6 at Ijesa. The use of LLINs at Ijesa (PN 2.0) and Irolu (PN 3.0) reduced the risk of malaria transmission by close to 75 and 97% respectively compared to the UTC village (Table 2).

**Table 4:** **Blood feeding preference and sporozoite rates for *An. gambiae s.s.*** **collected from the three study villages, at baseline and monthly mean during the 12-months post-intervention period**

**Mosquito population**	**Treatment**	**Time of testing**	**Blood positivity rate (%)**					**Sporozoite positivity rate**		
			**No. tested**	**Human only**	**Cattle only**	**Other**	**p value**	**No. tested**	**Positive (%)**	**p value**
**Ilara**	UTC	Baseline	295	82.7	13.4	3.4	0.558	568	1.76	0.709
		Post-intervention	1500	81.1	13.0	10.5		4345	2.09	
**Irolu**	PN 3.0	Baseline	350	80.9	17.1	2.0	0.042	702	2.14	0.022
		Post-intervention	27	29.6	70.4	0		573	0.87	
**Ijesa**	PN 2.0	Baseline	360	85.0	13.9	1.1	0.091	745	3.08	0.832
		Post-intervention	548	60.0	36.0	4.0		2415	2.81	

p value < 0.05 indicates a significant difference between baseline and post-intervention monthly mean.

#### Net use and performance

Data from the baseline survey showed that 52-58% of respondents from the three villages attested to the use of aerosols as the main practice for controlling mosquito bites. The use of LLINs was not a common practice in the three villages.

The post intervention follow up showed that three months after the commencement of the study, about 75% of the 150 households in the UTC village had removed the untreated nets from their beds. The reasons given by all respondents were that the untreated nets provided no protection against mosquito bites and none of them had the nets by the 12^th^ month following distribution. In contrast, all of the 137 households with PN 3.0 and 147 with PN 2.0 still had the net mounted in their room 12 months after net distribution. However, when individuals were asked whether they were still sleeping regularly under the LLINs, 98 and 84% of households in PN 3.0 and PN 2.0 respectively still used the nets with a significant difference in net usage by village (Fisher’s exact test, p = 0.032).

Physical examination of nets after 12 months of field use showed that most of the PN 3.0 (98.5%) and PN 2.0 (93.8%) were in good condition, having no holes. Only two PN 3.0 had 3–5 holes (mean diameter = 2.5 cm) while 10 PN 2.0 had 3–8 holes (mean diameter = 2.8 cm).

Skin irritation was the main side effect reported by 19.7% and 16.3% of households using PN 3.0 and PN 2.0 respectively. A similar proportion of people (about 15%) from both LLIN villages also reported sneezing. Overall, a significantly higher proportion of people using PN 3.0 (92.7%) versus PN 2.0 village (74.1%) indicated that the intervention was beneficial (p = 0.036). The descriptive data from the focus group discussion indicated this was because PN 3.0 was perceived to reduce the number of mosquitoes, bed bugs and cockroaches during the study compared to nets previously distributed in the area.

## Discussion

This study compared the efficacy of two LLINs at two individual villages with untreated nets at another village. An obvious limitation of this study is the lack of replication, as only one village per net-type was used, however, the similarity in baseline entomological indices, mosquito control practices and demographic characteristics of the villages in the study area in part explains the reason for employing this study design.

*Anopheles gambiae s.s*., the major malaria vector in the area, occurred in large numbers for 6–7 months, mainly during the wet season as earlier reported [14]. On average, fewer mosquitoes were found from November until February in the UTC control village. This seasonal abundance pattern would be expected to be similar for other villages in the area where transmission of *Plasmodium falciparum* continues to occur mainly in the wet season, although more control villages would be required to verify this. Seasonality in vector densities was clearly evident in the UTC village, partially evident in the PN 2.0 village but not evident in the PN 3.0 village, largely due to the consistently low vector densities post-intervention in the PN 3.0 village.

Pyrethroid and DDT resistance were found in the three villages during the baseline and post intervention surveys with a similar level of phenotypic resistance in both pre- and post-intervention periods. The *kdr* mutation was the sole resistance mechanism detected at Ilara, with *kdr* + metabolic p450-based resistance mechanisms detected at Irolu and Ijesa. The presence of both *kdr* + metabolic p450-based resistance mechanisms in the mosquito population from this study area alludes to an earlier notion of the presence of multiple pyrethroid resistance mechanisms in the malaria vector *An. gambiae s.s* in Nigeria [8]. However, in spite of the presence of these resistance mechanisms, the use of PN 3.0 at Irolu significantly reduced not only the mosquito density per house, but also the blood feeding and parity rates compared to the PN 2.0 and UTC control villages. This decrease was consistent during the twelve months following PN 3.0 distribution. Aside from the ‘mass killing’ effect of *Anopheles* caused by PN 3.0, the low parity rate in the PN 3.0 compared to the PN 2.0 village is an indication of the reduction in the parous population and the resultant reduction in risk of malaria transmission, as reflected in the appreciable reduction in the post intervention entomological inoculation rates. This indicates that PN 3.0 may have resulted in a reduced mosquito life span and survival rate. The results also showed a shift in host preference after PN 3.0 distribution with a significant number of mosquitoes feeding on cattle in contrast to humans during the baseline period. This is a surprising finding, given the strong human feeding preference of *An. gambiae s.s.*, and could be a consequence of the lower sample size as there were far fewer mosquitoes to test during the post-intervention period. It could be that the use of PN 3.0 induced changes in the endophilic tendencies in *An. gambiae* populations, such that a higher level of excito-repellency occurred that may induce outdoor biting behaviour. This effect coupled with the high mosquito mortality due to the use of PN 3.0, may result in outdoor locations becoming an important venue for host-seeking *An. gambiae s.s* during the use of PN 3.0.

Analysis of chemical content of nets of LLINs showed a marked loss of PBO content from PermaNet® 3.0 at 6 months post-distribution. However, there was no change in PBO content evident between 6 and 12 months post-distribution. The rapid initial loss may be due to an accumulation of PBO on the surface of new nets, which is rapidly depleted through washing, handling and evaporation at the onset of usage. It may also indicate stabilization of the PBO migration rate throughout the polymer during early usage leading to minimal loss over the subsequent 6 months period. Related studies with permethrin-PBO combination LLIN (Olyset® Plus) in Benin and Cameroon [29] showed that after just three washes there was a loss in killing effect against resistant strains of *An. gambiae* from Benin (92% before and 56% after washing) and Cameroon (98% before and 69% after washing), also indicating rapid loss of PBO in permethrin-PBO combination [30]. Regardless of the initial depletion of PBO from PermaNet® 3.0, this combination LLIN exhibited enhanced efficacy when compared to the deltamethrin-only PermaNet® 2.0 over the 12 month study duration. To further evaluate the migration dynamics and loss rates of PBO and pyrethroids from combination LLINs during field usage, extended field studies would need to be conducted.

Observations from the questionnaire surveys yielded insight into human behaviour in the study area. Human activities outside the home into the late evening hours are not common in the area. Therefore, with mosquitoes either reluctant to enter PN 3.0 households, or more likely to leave, and the absence of humans outdoors when the biting of *An. gambiae s.s* is at its peak, a considerable amount of *An. gambiae* s.s. blood meals were taken from alternative hosts such as cattle, as indicated in the post intervention blood feeding data from the PN 3.0 village. This is clearly a contributing factor to the reduction in malaria transmitting mosquitoes observed from the PN 3.0 village in the post-intervention period. Additionally, marginally fewer PN 3.0 had holes than PN 2.0, despite higher reported usage rates of PN 3.0. The greater proportion of householders reporting benefits of PN 3.0 compared to PN 2.0 is also consistent with studies conducted previously in Nigeria [12].

Overall, the results showed a significant impact of PermaNet® 3.0 on the mosquito population relative to that observed at the PermaNet® 2.0 village. This study is limited by the lack of replicates of each treatment arm, and the single point mosquito collection made at baseline. However, the results are consistent with similar work carried out in an area with *kdr* + metabolic based resistance mechanisms in malaria vector populations at other sites in Nigeria [12] and elsewhere in Africa [29-32] and supports increasing evidence indicating a reduction in efficacy of pyrethroid only LLINs against pyrethroid resistant malaria vectors [33,34].

## Conclusion

The presence of pyrethroid resistant vector populations permitted the assessment of the impact of PN 3.0 on mass community protection against pyrethroid resistant malaria vectors. The use of PN 3.0 significantly reduced mosquito densities per house, which was coupled with an observation of changes in the bloodmeal origin, sporozoite rate and parity rate in the *An. gambiae* population resulting in a significant reduction in transmission indices. The trial confirmed that in the presence of *kdr* plus P450-based metabolic resistance, there was an increased efficacy of PN 3.0 compared to the pyrethroid-only LLIN (PN 2.0). The data presented in this study along with previous work in Nigeria suggests that the use of PN 3.0 will contribute towards a reduction in malaria transmission over time when compared to existing pyrethroid-only LLINs in areas with P450-based pyrethroid metabolic resistance.

### Ethical approval

The study was approved by the Institutional Ethics Review Committee of the Nigerian Institute of Medical Research. All households in the three villages indicated their willingness to participate in the study and gave written consent.

## Competing interests

The authors declared that they have no competing interests. Although the study was funded by Vestergaard Frandsen, the findings described in this manuscript are those of the authors and do not necessarily reflect views of Vestergaard Frandsen.

## Authors’ contributions

TSA designed the study protocols and drafted the paper. AOA, IOO and AOO coordinated and supervised the field collections, JBO coordinated laboratory work and analysed the data, CNA conceived the study and participated in the design. All authors read and approved the final version of the manuscript.
